# Effects of *Salvia officinalis* L. (common sage) leaves tea on insulin resistance, lipid profile, and oxidative stress in rats with polycystic ovary: An experimental study

**Published:** 2020

**Authors:** Mahnaz Ghowsi, Namdar Yousofvand, Saman Moradi

**Affiliations:** 1 *Department of Biology, Faculty of Sciences, Razi University, Kermanshah, Iran*

**Keywords:** Salvia officinalis L., Polycystic ovary syndrome, Lipid profile, Insulin resistance, Oxidative stress

## Abstract

**Objective:**

Oxidative stress conditions and metabolic complications are common among polycystic ovary syndrome (PCOS) patients. There are various reports about hypoglycemic and antioxidant effects of *Salvia officinalis *L. (common sage). This study evaluated the possible medicinal effects of sage tea drinking on oxidative status, lipid profile, and insulin resistance in rats with testosterone-induced PCOS.

**Materials and Methods:**

Eighteen immature female Wistar rats (21-day old) were divided into 3 groups: 1) The Control group (n=6) that received no treatment. 2) The PCOS group (n=6) that received testosterone enanthate 10 mg/kg BW for 35 days subcutaneously. (3) The PCOS -sage tea group (n=6) to which after induction of PCOS by injection of testosterone enanthate, the sage tea was administered as a replacement of water for 14 days. The beverages were refreshed every day. The serum levels of total antioxidant capacity (TAC), malondialdehyde (MDA), glucose, insulin, HDL-C, total cholesterol, LDL-C, VLDL-C, total triglycerides, and atherogenic index were measured.

**Results:**

Sage tea consumption increased serum TAC and decreased serum HDL-C, glucose, total cholesterol, LDL-C, and atherogenic index levels but it did not change the levels of MDA, insulin, total triglycerides, and VLDL-C.

**Conclusion:**

Results suggested that sage tea consumption may influence the oxidative status and reduce the blood glucose and atherogenic index and may have cardiovascular protective effects in PCOS women.

## Introduction

Polycystic ovary syndrome (PCOS) is a common heterogeneous endocrine disorder that affects 6-10% of women in premenopausal ages and may be associated with metabolic disorders such as hyperglycemia, insulin resistance, obesity, and increased risk for metabolic syndrome and type 2 diabetes mellitus (Rojas et al., 2014 ▶).

Some studies suggest that oxidative stress play an important role in the pathophysiology of PCOS and this condition may increase the cardio-metabolic risk and insulin resistance in these patients (Desai et al., 2014 ▶; Rojas et al., 2014 ▶). Also, it seems that oxidative stress be an important factor in the induction of hyperandrogenism in PCOS (Duleba and Dokras, 2012 ▶). Nowadays, there is growing interest in the use of herbs for treatment of metabolic disorders that are commonly associated with PCOS. *Salvia officinalis *L. (common sage) from the family *Lamiaceae* is a medicinal plant that is cultivated in various countries such as Canada, the USA, Turkey, India, Japan, Indonesia (Java), Tanzania, South Africa, Antilles, Brazil, Australia, New Zealand, and Iran. Some studies reported that *S. officinalis *L. is an important antioxidant, anti-diabetic, anti-inflammatory and anti-microbial agent and may have protective effects against cardiovascular diseases and cancer (Grdiša et al., 2015 ▶). Some studies indicated that some parts of the plant such as leaves and branches are rich in phenolic components that have antioxidant effects. The most important phenolic compounds that are found in this plant are phenolic acids such as caffeic, vanillic, ferulic, and rosmarinic acids, flavonoids such as luteolin, apigenin, and quercetin, as well as α- and β-thujone, 1, 8-cineole, camphor, carnosic acid, carnosol, rosmadial, manool, and volatile substances (Shan et al., 2005 ▶; Jakovljevi´c et al., 2019 ▶). Various reports showed that the phenolic and flavonoid compounds, terpenoids, and coumarins are responsible for the antidiabetic, antioxidant, and free radical scavenging effects of medicinal plants (Hamidpour et al., 2014 ▶; Mehdizadeh et al., 2018 ▶). Various studies showed that oxidative stress and free radicals are involved in the development of many disorders such as diabetes and cardiovascular diseases and control of oxidative stress have protective effects against these conditions (Eidi et al., 2006 ▶; Yadav and Mukundan, 2011 ▶). It was reported that drinking of sage tea (infusion) improved oxidative status in the livers of rat and mice (Lima et al., 2005 ▶). *S. officinalis* L. has been consumed as a traditional herb for diabetes treatment and it was reported that it has glucose lowering effects (Christensen et al., 2010 ▶). Also, it was reported that an aqueous extract of sage had insulin-like activities (Christensen et al., 2010 ▶). As people usually consume sage as tea (Salah et al., 2016 ▶), this study evaluated effects of drinking *S. officinalis* L. leaves tea on total antioxidant capacity (TAC) and malondialdehyde (MDA) levels as two oxidative stress biomarkers, insulin resistance, and lipid profile in a rat model of PCOS. 

## Materials and Methods

**Collection of sage and preparation of the tea**

Green and fresh sage plants were purchased from a farm at Islam Abad Gharb and identified by faculty members of Razi University, Kermanshah, Iran. Plant leaves were dried under shade. Then, they were powdered manually and stored at room temperature. The tea of sage was made according to Lima et al. instruction as follows: 2 g of dried plant leaves was added to 150 ml boiling water and was soaked for 5 min. Each ml from this herbal tea solution produced 3.5 ± 0.1 mg extract dry weight in which there were rosmarinic acid (362 µg/ml infusion) and luteolin 7-glucoside (115.3 µg/ml infusion) as major phenolic compounds, and 1, 8-cineole, cis-thujone, trans-thujone, camphor, and borneol as the major volatile components (4.8 µg/ml infusion) (Lima et al., 2005). The beverages were refreshed every day. 

**Animals and experimental design**

This study was carried out in eighteen immatures (21-day old) female Albino Wistar strain rats (*Rattus norvegicus*) and till the end of the experiments, all animals survived. The animals were divided into 3 groups: I) The Control group (n=6) received no treatment. II) The PCOS group (n=6) included polycystic ovary rats that subcutaneously received testosterone enanthate 10 mg/kg for 35 days (Beloosesky et al., 2004 ▶). III) The PCOS-sage tea group (n=6) to which, after injection of testosterone enanthate 10 mg/kg for 35 days, sage tea was given according to Lima et al. (Lima et al., 2005 ▶). The sage tea was given to the animals as a replacement for drinking water for 14 days.

The amounts of water and sage tea that were drunk by rats were not significantly different among the groups. Animals had free access to food and water or the sage tea. All the protocols and animal treatments were conducted according to the approvals of Institutional Animal Care and Ethical Committee of Biological Sciences of Razi University and all efforts were made to minimize the number of animals that were used.

At the end of the experiment, the animals were anesthetized by a combination of combination of ketamine (50 mg/kg) and xylazine (7 mg/kg) and ovaries and blood samples were obtained. The blood samples were left to coagulate at room temperature and centrifuged at 3000 rpm for 20 min. The serum samples were prepared and kept at -30ºC until biochemical analysis. The polycystic ovary phenotype was well established as shown by the results of evaluation of slides prepared from ovary sections and measurement of testosterone concentrations in the serum samples.

**Biochemical assessments**

The fasting serum levels of insulin were determined by rat ELISA kit (Mercodia AB, Sylveniusgatan 8A, SE-754 50 Uppsala, Sweden) and the serum testosterone concentration was measured by radio immunoassay. The fasting serum levels of glucose were measured and the HOMA-IR HOMA-IS levels were determined according to the following formulas:

HOMA-IR= fasting insulin × fasting glucose/405

HOMA-IS= 10000/fasting insulin × fasting glucose (Aref et al., 2013 ▶).

Serum total triglycerides, LDL-C, and HDL-C were measured. The serum levels of VLDL-C, and total cholesterol, and atherogenic index (AI) were calculated by the following formulas: 

VLDL- cholesterol concentration= Triglycerides/5

Total cholesterol= (HDL-C) + (Triglycerides/5) + (LDL-C) 

AI= (total cholesterol- HDL-C)/ HDL-C (Hassan et al., 2011 ▶).

**Measurement of TAC and MDA levels in serum**

A commercial kit was used for measurement of antioxidant capacity on the basis of the oxidation/reduction colorimetric assay (ZellBio GmbH, Wurttemberg, Germany) according to the manufacturer’s instructions. TAC amount was considered the amount of antioxidant in the sample as compared with ascorbic acid as a standard. The serum MDA levels were determined by an Agilent technology 1200 series HPLC system (Agilent Corp., Germany) with EC 250⁄4.6 Nucleodur 100-5 C18ec column (Macherey-Nagel, Duren, Germany). A sample of serum or 1, 1, 3, 3- tetraethoxypropane (TEP) standard (50 µl of a stock standard solution containing 5 µmol/l TEP in 40% ethanol solution) was added to 50 µl butylated hydroxytoluene (BHT) (0.05% v⁄v BHT in ethanol), 400 µl H_3_PO_4_ and 100 µl 2-thiobarbituric acid (TBA) (42 mmol/l in 0.44 mol/l H_3_PO_4_) and it was kept at 100ºC for 1 hr. Then, samples were chilled for 10 min. Then, the MDA-TBA complex was removed from the mixture using n-butanol (250 µl). The samples were mixed for 5 min and centrifuged for 3 min at 14000 rpm to separate the two phases. Aliquots of 100 µl were extracted from the n-butanol layer of each sample. To measure MDA levels, 20 µl of the n-butanol extract was injected into an HPLC reverse-phase column using a mixture of methanol and 50 mmol/l phosphate buffer, pH 6.7 (40⁄60, v⁄v (%)) as the mobile phase. The MDA peak at 553 nm, excitation 515 nm detected. To confirm the values of the eluted MDA a commercial standard was used and its quantity was determined by peak area using agilent technologies 1200 series software (Asefi et al., 2012). BHT, TBA, and TEP were purchased from Sigma-Aldrich Co. (St Louis, MO, U.S.A.) and the other materials were purchased from Merck (Darmstadt, Germany).

**Statistical analysis**

Results were analyzed by SPSS (version 16) software using one-way ANOVA followed by *post hoc* Tukey’s test analysis. Data are shown as mean±SEM for each group. In all cases, p values less than 0.05 were considered statistically significant.

## Results

**Figure 1:**
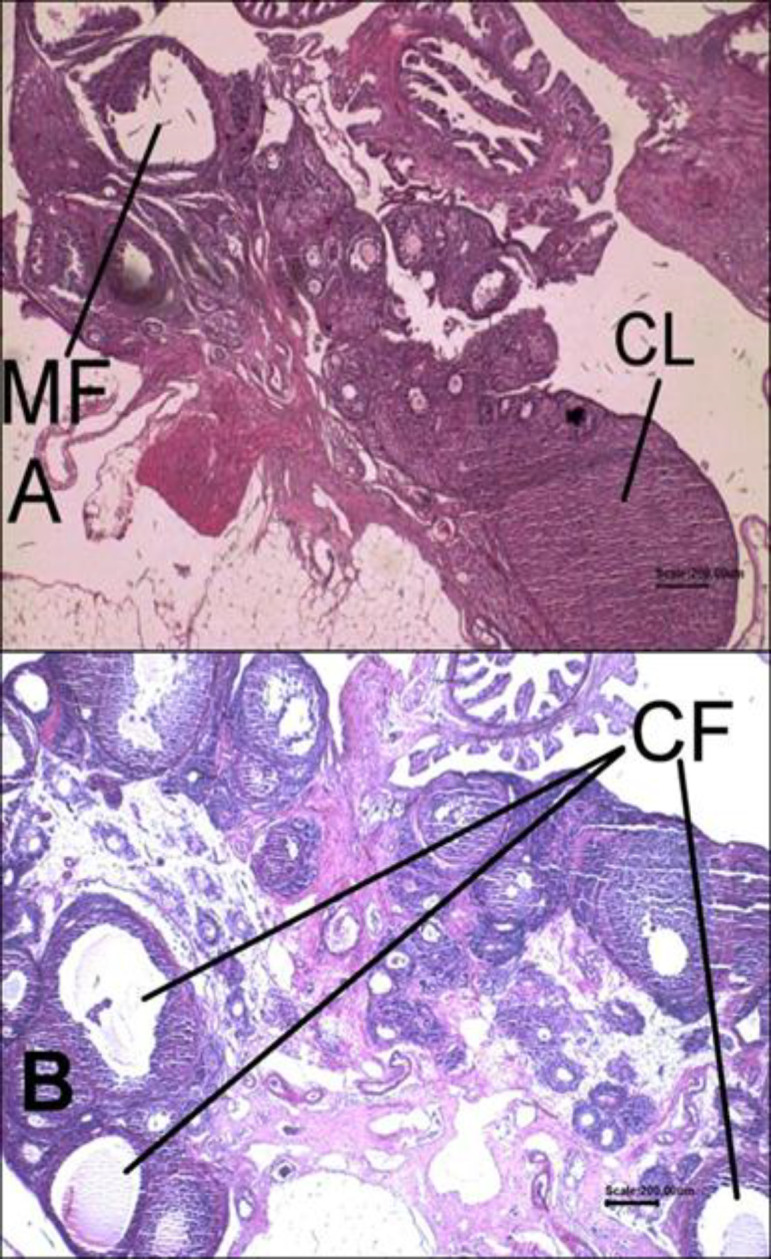
Histological results of hematoxylin and eosin (H&E)-stained sections. The sections of animals’ ovaries in the control group (A) and in the PCOS group (B). MF: Mature follicle; CL: Corpus luteum; CF: Cystic follicle (Magnification X40)

The results showed that the ovaries of control rats had normal histomorphology of the corpus luteum (Figure 1A), but in the PCOS group, the ovaries of rats were polycystic (Figure 1B) and testosterone concentrations were higher than those of the control group (p=0.000) (Table 1). The mean of testosterone levels in the PCOS-sage tea group was more than those of control rats (p=0.000) (Table 1).

The analysis of results revealed that in the PCOS group, glucose concentrations were higher than those of control rats (p=0.001) and in the PCOS -sage tea group, the mean of glucose concentrations were decreased when compared to the PCOS group (p=0.012). The mean of insulin levels were not significantly different between the PCOS group and the PCOS group (p=0.561). Also, insulin concentrations in the PCOS group were not statistically different from insulin levels in the controls (p=0.455). In the PCOS group, the HOMA-IR were higher than those of in the control group (p=0.034) but, this parameter in the PCOS group and the PCOS-sage tea group were similar (p=0.865) (Table 1). 

The means of HOMA-IS levels in the PCOS group (p=0.007) and the PCOS-sage tea (p=0.036) were significantly decreased compared to the control group. As presented in Table 1, the β-cell function levels in the PCOS-sage tea were significantly higher than those of the control group (p=0.039). The levels of this parameter were not significantly different between PCOS group and the control group (p=0.592) (Table 1).

**Table 1:** The levels of testosterone, insulin, glucose, HOMA-IR, and HOMA-IS in rats of PCOS, PCOS-sage tea, and control groups

**Group**	**Control **	**PCOS group **	**PCOS-sage tea **
**Testosterone (ng/ml)**	0.49±0.0048	2.3±0.05774	2.4±0.1581
**Insulin**	2.484±0.0171	3.103±0.3951	3.629±0.4736
**Glucose (mg/dl)**	88.07±3.599	130.1±8.311**	99.81±6.587≠
**HOMA-IR**	0.5383±0.02227	0.9928±0.1314*	0.9091±0.1478
**HOMA-IS**	46.12±1.988	26.89±3.315**	31.34±5.260*

Data are expressed as mean±SEM (n=6).^ *^p<0.05 versus the control group; ^**^p<0.01 versus the control group; and^ ≠^p<0.05 versus the PCOS group. HOMA-IS: Homeostasis model assessment for insulin sensitivity; and HOMA-IR: Homeostasis model assessment for insulin resistance.

**Figure 2:**
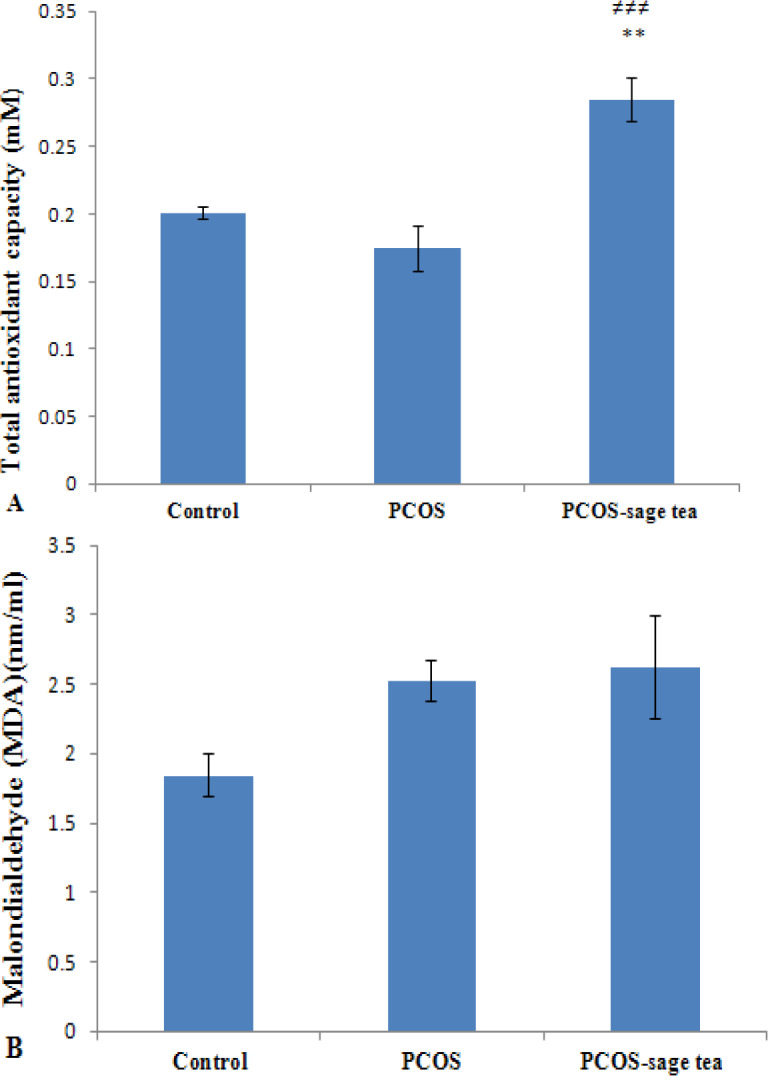
The levels of total antioxidant capacity (TAC) (A) and malondialdehyde (MDA) (B) in the serum samples of the control, PCOS and PCOS-sage tea groups. Data are expressed as mean±SEM (n=6). ^**^p<0.01 vs. control group; and^ ≠≠≠^p<0.001 vs. PCOS group

Our results showed that the mean of total antioxidant capacity (TAC) levels in the PCOS-sage tea group (n=6) were higher than those of the PCOS group (n=6) (p=0.000) (Figure 2A). Besides, in the PCOS-sage tea group, TAC levels were significantly higher than the control rats (n=6) (p=0.001) (Figure 2A). There were no significant differences in the mean of serum MDA levels between the PCOS and PCOS-sage tea group (p=0.956) (Figure 2B).

MDA concentrations in the PCOS group were not statistically different those of the controls (p=0.160) (Figure 2B).

As indicated in the Table 2, the levels of VLDL-C and total triglycerides were not significantly different between PCOS group and control group (p=0.88 and p=0.9, respectively) (Table 2). Also, VLDL-C and total triglycerides levels in the PCOS-sage tea group were similar to those of the PCOS group (p=0.17 and p=0.27) (Table 2).

The levels of LDL-C in the PCOS group were higher than the controls (p=0.000) and the levels of this parameter in the PCOS-sage tea were lower than the PCOS group (p=0.000) **(**Table 2). HDL-C levels in the PCOS and PCOS-sage tea were significantly lower than those of the control group (p=0.000). HDL-C concentrations in the PCOS-sage tea were significantly lower than those of the PCOS group (p=0.025) (Table 2). Total cholesterol levels in the PCOS group (p=0.026) were lower than those of the control group. Total cholesterol levels in the PCOS-sage tea were lower than the control (p=0.000) and PCOS group (p=0.002) (Table 2). 

**Table 2:** Serum levels of LDL-C, VLDL-C, HDL-C, total cholesterol, total triglycerides in the control, PCOS, and PCOS -sage tea groups

**Group**	**Control **	**PCOS group**	**PCOS-sage tea **
**VLDL-C (mg/dl)**	9.670±0.80	10.14±0.86	8.267±0.24
**LDL-C (mg/dl)**	5.667±0.21	8.000±0.26^***^	6.000±0.26^≠ ≠ ≠^
**HDL-C (mg/dl)**	35.00±0.36	28.00±0.26^***^	25.80±0.79^≠***^
**Total triglycerides (mg/dl)**	48.34±4.01	50.71±4.31	41.33±1.22
**Total cholesterol (mg/dl)**	50.34±1.10	46.14±0.94^*^	40.07±0.98^***≠ ≠^

Data are expressed as mean±SEM (n=6).^ *^p<0.05 versus the control group; ^***^p<0.001 versus the control group;^ ≠ ≠ ≠^p<0.001 versus the PCOS group; and ^≠ ≠^p<0.01 versus PCOS group.

**Figure 3:**
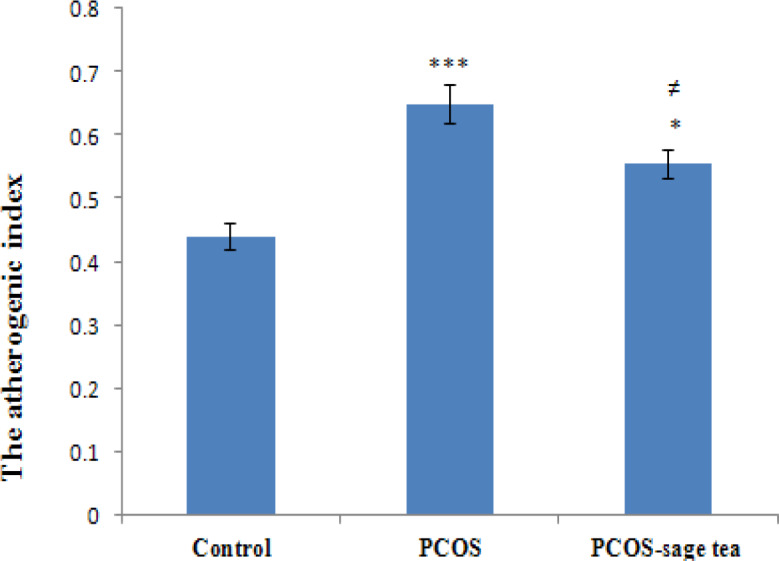
The atherogenic index levels in the control, PCOS, and PCOS-sage tea groups. Data are expressed as mean±SEM (n=6). ^*^p<0.05 vs. control group; ^***^p<0.001 vs. control group; and^ ≠^p<0.05 vs. PCOS group

In the PCOS group, the atherogenic index levels were higher than the control group (p=0.000) and in the PCOS-sage tea group, the atherogenic index levels were decreased compared to the PCOS (p=0.043) and control group (p=0.013) (Figure 3). 

## Discussion

Nowadays, there is an enormous interest in the use of herbal remedies for treatment of metabolic disorders, worldwide. Because of the presence of bioactive compounds like phenolic acids (caffeic acid, vanillic acid, ferulic acid, and rosmarinic acid) and flavonoids (luteolin, apigenin, and quercetin) that can act through different biological ways, sage has been used for treatment of different health conditions (Mahdizadeh et al., 2018 ▶; Jakovljevi´c et al., 2019 ▶). In the present article, we investigated possible antioxidant, anti-diabetic, and lipid-lowering effects of *Salvia officinalis* (sage) tea in the testosterone-induced polycystic ovary rats that were treated with sage tea for two weeks. Our results showed that the ovaries of animals in the PCOS group were polycystic and no corpus luteum was observed in the ovary sections and the testosterone concentrations were significantly higher than the control group. These results indicated that the features of PCOS were developed by injection of testosterone enanthate in the animals. 

After 14 days of treatment of polycystic ovary rats with sage tea, a significant decrease in the fasting serum glucose levels was observed. This finding is in accordance with the results of the study done by khedher et al. that showed that treatment of high-fat diet mice with low dose methanol extract of *S. officinalis* leaf for two weeks, improved tissue insulin sensitivity and decreased plasma insulin concentrations in response to glucose load (khedher et al., 2018). Similarly, the finding of the study done by Lima et al. showed that replacing water with sage tea for 14 days decreased plasma glucose levels in the normal mice (Lima et al., 2006 ▶). 

The antidiabetic effects of *S. officinalis* may be due to inhibition of the intestinal maltase and sucrase enzymes (Gayathri and Gayathri, 2016 ▶). It was shown that aerial parts of *Saliva genus *species usually contain flavonoids like glycoside kaempferol with hypoglycemic effect and cause increases in the glucose utilization by muscles in rats (Asadi et al., 2010 ▶). Eidi et al. study done in streptozotocin induced-diabetic rats showed that consumption of *Salvia* tea can inhibit gluconeogenesis (Eidi et al., 2005 ▶). Some mechanisms suggested for antidiabetic actions of *Salvia* species extracts are as follows: activation of pancreatic beta cells, increment of insulin sensitivity and peripheral utilization of glucose, inhibition of insulinase enzyme, reduction of glycogenolysis, decreases in the glucose absorption from intestine, and increment of the synthesis of glucose in the liver (Mahdizadeh et al., 2018 ▶). It was demonstrated that flavonoids particularly quercetin, have antidiabetic effects (Mahdizadeh et al., 2018 ▶). Also, it was demonstrated that a decrease in the fasting glucose level is a sign of decreased liver gluconeogenesis and/or liver glycogenolysis (Lima et al., 2006 ▶). Thus, these results may suggest that sage tea decreases the production of glucose by the liver. Our results suggested that *S. officinalis *L*.* tea may be a suitable agent for reduction of blood glucose levels in PCOS patients that have insulin resistance and are at risk of type 2 diabetes. However, in the present study, insulin resistance and insulin sensitivity indices were not improved by sage tea treatment. These findings are in contrast with those of Khedher et al. who reported that treatment with methanolic extract of sage for three weeks, decreased the HOMA-IR index (Khedher et al., 2018). Also, in the present study, treatment of polycystic ovary animals by sage tea did not alter insulin levels. This result is similar to results of Eidi et al. study that showed that injection of the methanolic extract of sage in the fasted STZ-diabetic rats did not affect insulin release (Eidi et al., 2005 ▶). 

In the present study, treatment of polycystic ovary rats by sage tea significantly increased serum TAC levels, but did not change the serum MDA (a product of lipid peroxidation) levels. These findings about TAC were in line with a study that showed that replacement of drinking water with sage tea improved the antioxidant status in the rats (Lima et al., 2005 ▶). Our results about MDA are against the finding of a previous study done by Khattab et al. that showed that treatment of the diabetic rats with sage significantly reduced blood MDA levels (Khattab et al., 2012 ▶). Previous studies showed that sage acts as a scavenger of free radicals (Iuvone et al., 2006 ▶; Bulku et al., 2010 ▶).

Sage has phenolic compounds with antioxidant effects such as carnosic acid and rosmarinic acid. The amount of rosmarinic acid in plant extracts prepared from different species of the genus of *Saliva* is between 28 and 64 µg/mg (Mahdizadeh et al., 2018 ▶). Various studies showed that the carnosic and rosmaric acids in sage have antioxidant effects and protect from oxidative stress ((Khattab et al., 2012 ▶; Grdiša et al., 2015 ▶). In addition, salvianolic acid that is the dimmer of rosmaric acid acts as a potent antioxidant and free radical scavenger (Lu and Foo, 2001 ▶). It is likely that the increase in the serum TAC levels in the present study might be due to the presence of these antioxidant components in the sage tea. 

Alterations in glucose and lipid profile are related to risk for diabetes, metabolic syndrome, and cardiovascular diseases (Hernandez-Saavedra et al., 2016 ▶). The results of our study showed that in polycystic ovary rats, LDL-C were increased and HDL-C levels were decreased when compared to the control rats. These findings are in accordance with the results of previous reports that showed that in the PCOS women, the levels of LDL-C are increased and the levels of HDL-C are decreased (Wild et al., 1985 ▶; Legro et al., 2001 ▶; Yilmaz et al., 2005 ▶). In the present study, the serum LDL-C and total cholesterol levels were decreased in the polycystic ovary rats treated with sage tea in comparison to polycystic ovary rats that did not receive sage. In this regard, Lima et al. showed that drinking of a *S. officinalis* infusion decreased the levels of cholesterol and triglycerides in the blood (Lima et al., 2005 ▶). Also, another study reported that treatment of diabetic rats with sage (oral administration) reduced serum levels of LDL-C, VLDL-C, total cholesterol, and triacylglycerol (Khattab et al., 2012 ▶). Similarly, our study showed that treatment of polycystic ovary rats with sage tea decreased the serum LDL-C and total cholesterol levels. However, in our study, the levels of VLDL-C and total triglycerides did not alter significantly. Hernandez-Saavedra et al. showed that consumption of the sage infusion for 12 weeks reduced total cholesterol, triglycerides, low-density lipoprotein in rats (Hernandez-Saavedra et al., 2016 ▶). Also, in a study by Sá et al., six women consumed 600 ml of sage infusion per day for 4 weeks. Then, they did not drink sage tea for 2 weeks and their blood glucose and lipid profile were assessed. Their results showed that sage tea consumption has no effect on the glucose, but decreased LDL and the total cholesterol levels and increased HDL levels (Sá et al., 2009 ▶). 

These findings may be due to the inhibitory effects of *S. officinalis* on cholesterol biosynthesis (Moram, 2001 ▶; Eidi and Eidi, 2009 ▶). Also, it may be due to a monoterpene thujone that is one of the ingredients of *S. officinalis*. Thujone has lowering effects on cholesterol and triglyceride levels (Khattab et al., 2012 ▶). In this regard, some studies showed that sage extracts caused PPARγ activation. This factor is an important regulator of genes involved in lipid and glucose metabolism and its activation results in improvement of HDL/LDL ratio and reduction of adipose tissue (Christensen et al., 2010 ▶). In our study, treatment with sage did not change triglyceride and VLDL levels. Analysis of results of the present study showed that treatment of polycystic ovarian rats with sage tea significantly decreased the atherogenic index levels. In line with our findings, a study done in humans showed that consumption of sage tea (300 ml, twice daily) improved lipid profile and antioxidant defense without having side effects and hepatotoxicity (Carla et al., 2009). These finding suggest that drinking sage tea may improve the metabolic complications usually associated with PCOS. 

In summary, our results showed that *S. officinalis *L. (common sage) tea consumption decreased levels of serum glucose, LDL-C, and total cholesterol, and atherogenic index in polycystic ovary rats. Considering the results of current study, sage tea usage may be useful in improvement of metabolic disorders and dyslipidemia that are commonly associated with PCOS and the plant may have protective effects against cardiovascular diseases and oxidative stress in PCOS patients. 
